# Strategizing global health governance: unpacking opportunities and challenges for least developed nations within the WHO pandemic treaty framework

**DOI:** 10.3389/fpubh.2023.1321125

**Published:** 2023-11-06

**Authors:** Shisong Jiang, Emmanuel Kumah

**Affiliations:** ^1^School of Law, Chongqing University, Chongqing, China; ^2^Department of Health Administration and Education, Faculty of Science Education, University of Education, Winneba, Ghana

**Keywords:** pandemic treaty, global health, international cooperation, COVID-19, least developed nations

## Abstract

Exploring the intricacies of the proposed WHO pandemic treaty, this paper underscores its potential benefits and challenges for Least Developed Nations (LDNs) in the global health landscape. While the treaty could elevate LDNs’ access to vital resources, fortify health systems, and amplify their voice in global health governance, tangible challenges in safeguarding equitable access, protecting sovereignty, and ensuring compliance are illuminated. Concluding with targeted recommendations, the paper advocates for treaty revisions that assure resource access, safeguard LDNs’ autonomy, and foster capacity-building. In essence, the paper emphasizes the imperative of genuinely empowering LDNs, crafting a pandemic treaty that establishes a more equitable, resilient, and inclusive global health future.

## Introduction

The imperative for international cooperation, particularly in the crucible of global health crises such as pandemics, has garnered extensive discussion over the years (1, 2). Such cooperative endeavors are quintessential for a plethora of reasons, enveloping the mutual exchange of resources, acumen, and expertise; the orchestration of synchronized response strategies; and the mitigation of the multifaceted social and economic reverberations engendered by pandemics (3). The COVID-19 pandemic, which began in 2019, unveiled a multitude of frailties in the international community’s proficiency in adeptly navigating public health emergencies (4, 5). For instance, the contentiously dilatory response to the initial outbreak in Wuhan, China, accentuated a conspicuous absence of a robust early warning system, during which the virus permeated international borders in the ensuing weeks prior to its identification (6). The WHO-China Joint Mission report, promulgated in February 2020, acknowledged a conspicuous lapse in global preparedness, articulating that “[m]uch of the global community is not yet ready, in mindset and materially” (7). Numerous other formidable challenges and deficits emerged during the global response to the COVID-19 pandemic, inclusive of constrained access to resources, pronounced nationalistic tendencies, coordination tribulations, the dissemination of misinformation, and an exigency for a comprehensive overhaul of global health governance (4).

The delineated weaknesses highlighted the necessity for a more amalgamated and coordinated global approach (8). Consequently, during a Special Session of the World Health Assembly (WHA) on December 1, 2021, member states of the WHO instantiated an intergovernmental negotiating body (INB) to formulate and negotiate a convention, agreement, or other international instrument pertaining to pandemic prevention, preparedness, and response, colloquially referred to as the “Pandemic Treaty” (9). This treaty seeks to navigate a myriad of facets inherent in pandemic management, encompassing surveillance, data sharing, vaccine allocation, and ensuring access to indispensable medical supplies. The nascent draft of the treaty, dubbed the Zero Draft of WHO CA+, was published on February 1, 2023 (10), and deliberated upon during the INB’s fourth, fifth [which had requested the INB to prepare a “Bureau’s text” that was published in June 2023 for facilitating the work of the Drafting Group (11)], and sixth meetings, transpiring from February to July 2023 (12). The finalized document is anticipated to be presented at the 77th WHA in May 2024 (13).

Since the treaty was proposed in 2021, a spectrum of arguments, both in favor and opposition, have been advanced by a diverse confluence of world leaders and academic scholars [e.g., (3, 11–18)]. Engaging in this debate, and advocating for the pandemic treaty, we hone our focus on LDNs and explore the myriad ways in which the proposed treaty could be a harbinger of assistance for these countries during future outbreaks. In this endeavor, we firstly elucidate the circumstances of LDNs amidst pandemics, subsequently exploring the potential merits of the pandemic treaty for these countries, followed by an exploration of pivotal challenges and concerns that necessitate addressal to ensure the treaty’s efficacious and equitable implementation, and concluding with recommendations to fortify the impact of the treaty upon LDNs.

This paper proffers insights that are both critical and timely, nested within the ongoing discussion and negotiation surrounding the pandemic treaty. Primarily, accentuating the predicament of LDNs within the context of pandemics is paramount as it unveils the exigencies of a vulnerable population that warrants specialized assistance. Secondly, by concretely discussing how the proposed treaty could be a conduit of benefit for LDNs, the paper underscores the salience of ensuring that responses to pandemics are equitable and just, irrespective of a nation’s economic vitality. Thirdly, by elucidating the potential boons of the treaty for LDNs, the paper underscores the affirmative impact such a treaty could potentially yield upon these nations, thereby potentially serving as a compelling advocacy for its adoption and implementation. Fourthly, engaging in discussions pertaining to challenges and concerns related to the treaty’s efficacious and equitable implementation is paramount as it enables policymakers and stakeholders to foresee and strategically navigate potential impediments. Finally, proffering recommendations for enhancement posits a pragmatic methodology to augment the treaty’s efficacy, ensuring it tangibly benefits the most susceptible nations.

### The pandemic-induced vulnerabilities of LDNs

LDNs, also known as least developed countries (LDCs), designated as nations entwining low-income, substandard human development indicators, and inherent structural vulnerabilities (19), grapple with a myriad of pronounced challenges during pandemics (20, 21). These nations confront a particularly perturbing array of hardships that frequently magnify the repercussions of such health crises (22). A paramount obstacle pivots around the paucity of robust healthcare facilities (23). A substantial number of LDNs are beleaguered by an insufficiency of hospitals, clinics, and healthcare centers, thereby curtailing their ability to dispense adequate care amidst pandemics (22). This infrastructural inadequacy precipitates congested and overwhelmed healthcare settings, complicating the enactment of efficacious infection control measures and thereby amplifying the risk of unbridled disease transmission (24).

Moreover, LDNs perennially wrestle with the daunting task of recruiting and retaining proficient healthcare personnel (25). Deficiencies in the availability of doctors, nurses, and allied healthcare professionals truncate their capacity to mount an effective response to pandemics. For instance, the African continent, home to many LDNs, endures a stark scarcity of healthcare workers, showcasing a doctor-patient ratio that languishes significantly below global benchmarks (26). Such deficits invariably saddle the healthcare system, obstructing the provision of holistic care during pandemics.

In addition, encumbered by limited financial capacities, LDNs are habitually hamstrung in their ability to enact a comprehensive response to pandemics (27). The imposition of restrictive healthcare budgets culminates in a dearth of funding for indispensable medical supplies, equipment, and pharmaceuticals (28). These fiscal limitations not only jeopardize patient care but also attenuate the potential for the timely identification and surveillance of infectious diseases (29). Compounding these challenges, most LDNs experience elevated population densities and substantial informal settlements (30), wherein residents dwell in proximate quarters with restricted access to clean water and sanitation facilities (31). Such conditions catalyze the expeditious transmission of infectious diseases, a scenario vividly portrayed in the densely populated locales of India during the COVID-19 pandemic (32). Additionally, the scarcity of access to advanced medical technologies, encompassing diagnostic tools and life-saving apparatuses, perpetually delays the diagnosis of pandemics and impedes the delivery of critical care to afflicted individuals (33). This technological void also stifles the development and deployment of efficacious vaccination initiatives (34), contact tracing (35), and data monitoring systems (36), further exacerbating the challenges faced by LDNs in the context of global pandemics.

### Potential benefits of the pandemic treaty for LDNs

The proposed pandemic treaty, or the WHO CA+, aims to prevent pandemics, save lives, reduce disease burden, and protect livelihoods through strengthening the world’s capacities for pandemic prevention, preparedness, response, and recovery of health systems [(10), Article 3]. The treaty is guided by the principles of equity, human rights, and solidarity, and recognizes the sovereign rights of countries, the differences in levels of development among countries, and the existing relevant international instruments [(10), Article 4]. It, if effectively negotiated and implemented, could potentially offer several benefits to LDNs that have been disproportionately affected by the COVID-19 pandemic and may face similar challenges in future pandemics.

Firstly, the treaty holds the potential to greatly enhance LDNs’ access to critical pandemic-related products and technologies, an arena where they have faced chronic constraints. By planning for needs-based global supply chains and distribution networks for vaccines, therapeutics, diagnostics, and personal protective equipment [(10): Article 6], the treaty can help integrate LDNs into priority access and allocation mechanisms. This is pivotal, given LDNs’ limited domestic manufacturing capacities and heavy reliance on imports for these life-saving tools, as evidenced during COVID-19 where many LDNs had very low vaccination rates, with some countries administering less than 5 doses per 100 people, while advanced economies secured robust coverage (37). Beyond access, provisions to promote voluntary knowledge and technology transfers [(10): Article 7], including through coordination between originating and recipient countries, can seed localized skills, expertise, and infrastructure for producing pandemic countermeasures over the long term. Crucially, by providing policy space for intellectual property flexibilities [(10): Article 7.4], the treaty opens legal pathways for LDNs to gain affordable access to innovations through avenues like compulsory licensing without facing punitive actions. Taken together, these treaty components can equip LDNs with institutional frameworks, resources, and policy latitude to gain rapid access to pandemic tools both now and in the future.

Secondly, the treaty places health system resilience at the heart of preparedness, recognizing its fragility in LDNs. Provisions requiring context-specific planning to sustain capacities ranging from infrastructure to workforces [(10): Article 11], as well as protecting human rights [(10): Article 14] to address social determinants of health, provide an enabling blueprint for LDNs to deliver effective and equitable healthcare during outbreaks through resilient systems. Beyond infrastructure and training, this requires adequate financing – another arena where the treaty holds transformative potential through proposed instruments like solidarity funds and insurance schemes [(10): Article 19] that can inject greater budgetary predictability and sustainability into LDNs’ pandemic preparedness and response. Just as importantly, the treaty provides for collective governance mechanisms from global coordination councils [(10): Article 15] to whole-of-society approaches nationally [(10): Article 16], facilitating integrated planning, implementation, and monitoring with LDN involvement. Taken in totality, these provisions centered on health system resilience provide visible pathways for LDNs to move from fragmented and under-resourced responses to coordinated, rights-based pandemic management through robust national and international governance frameworks.

Thirdly, another major benefit lies in expanding LDNs’ equitable access to and participation in global pathogen and genomic sequencing data sharing, recognizing their frequently limited in-house surveillance, modeling, and research capacities. The treaty mandates timely sharing of pathogens and sequences [(10): Article 8], as well as establishing benefit-sharing systems to facilitate affordable LDN access to resulting innovations, like diagnostics, therapeutics, and vaccines [(10): Article 10]. For LDNs that have scarce epidemiological resources, provisions that channel shared data into early warning systems, risk assessments, and technical guidance from WHO and partners [(10): Articles 8, 9] can exponentially augment outbreak prediction, detection, and response. Additionally, by supporting technology transfers, training, and laboratory networks [(10): Article 7], the treaty provides means for LDNs to sustainably strengthen their own surveillance and research capabilities over time. Thus, the treaty provides both immediate and long-term avenues for LDNs to be structurally integrated into global pandemic vigilance and science cooperation as empowered actors.

Fourthly, while calling for whole-of-society pandemic literacy programs [(10): Article 17], the treaty spotlights the need to proactively counter misinformation through transparent, accessible public communications rooted in science. This is salient given limited health literacy and the proliferation of mis- and disinformation through digital channels can uniquely hinder outbreak response in LDN contexts. Beyond literacy, the treaty’s provisions requiring inclusive decision-making and multisectoral collaboration [(10): Articles 15, 16] provide opportunities to engage diverse community stakeholders in LDNs to build dialogue and trust between governments and citizens. Together, these provisions offer the potential to overcome barriers posed by misinformation and mistrust through purposeful communication and outreach strategies that meet LDN populations where they are.

Fifthly, the treaty brings considerations of climate change and environmental degradation [(10): Article 18] to the forefront of pandemic risk reduction, preparedness, and response for the first time in a global health instrument. As contexts facing disproportionate climate vulnerability and health system limitations, this focus on strengthening adaptive capacities is vital for LDNs. Codifying One Health approaches recognizing human, animal, and environmental interconnections provides an enabling framework for integrated surveillance and upstream interventions. Ensuring climate risks are factored into national action plans can also help institute resilience measures from disease early warning systems to health infrastructure safeguards. While broader in focus than pandemics alone, this emphasis on interlinkages provides urgency for LDNs to build systems that protect people amidst the compounding impacts of climate change through a shared agenda.

Lastly, the treaty lays substantial groundwork to bolster LDNs’ representation and participation in global health governance, providing venues to spotlight unique challenges and contributions while shaping collective action. Beyond affirming WHO’s coordination role [(10): Article 15], the treaty mandates inclusive decision-making processes [(10): Article 20] and establishes new bodies for stakeholder consultation on CA+ implementation [(10): Article 23]. These provisions formally integrate LDN perspectives and needs into the international legal architecture for outbreak preparedness and response for the first time. Furthermore, by planning regular treaty assessments and reviews [(10): Chapter VII], opportunities exist to continually refine cooperation and assistance provisions based on LDN experiences. Therefore, while imperfect, the treaty nonetheless provides an enabling framework for LDNs to gain a seat at the table and collectively chart a course towards more equitable pandemic health security worldwide.

In summary, while gaps and uncertainties remain in operationalization, the proposed pandemic treaty lays down important markers and pathways across multiple domains to consciously strengthen integration, empowerment, and support for LDNs in global outbreak preparedness and response. Above all, it provides the mandate and momentum for WHO and member states to work in solidarity towards addressing systemic inequities laid bare by COVID-19. If successfully adopted and faithfully implemented, it can spur action to provide LDNs with institutional capacities, policy space, knowledge assets, and collective action needed to safeguard lives and livelihoods against future global health emergencies.

### Challenges and concerns for LDNs in the pandemic treaty

Despite the potential opportunities crafted by the pandemic treaty, especially for LDNs, there exists a convoluted array of challenges that demand thorough analysis and strategic navigation. While the treaty heralds the promise of structured global response mechanisms, it simultaneously entwines LDNs into a complex web of disparities and hurdles if not meticulously adjusted and implemented (3, 38–40). Thus, it is vital to dissect the emerging challenges and potential pitfalls in the development, negotiation, and imminent implementation of the treaty, laying a foundation for exploring the nuanced issues in subsequent discussions.

A critical examination of the treaty unveils a pivotal concern: the absence of concrete and robust norms, especially those safeguarding equitable access to resources and information sharing (39). While the treaty gestures towards inclusivity and cooperation, it lacks stringent regulations and explicit norms to safeguard the rights and interests of LDNs (41), thereby casting a shadow on its potential to foster truly equitable global collaboration. This deficiency, as experts like Lawrence Gostin emphasize, not only blurs the obligations and incentives of high-income countries towards global health security contributions but also nudges the treaty towards the precipice of perpetuating pre-existing inequities and unpreparedness in global health governance (39), thereby underlining an urgent call for rigorous revision and fortification of its norms.

The intricacies of the treaty negotiations become further entwined when scrutinizing the principle of Common but Differentiated Responsibilities (CBDR) (42, 43). Initially woven into early drafts to champion equity by customizing obligations to align with each country’s capacity, CBDR has morphed into a contentious point, particularly between high-income nations and LDNs (44). This discord, illustrated by resistance from developed countries and a consequent dilution of CBDR in subsequent drafts, signifies a daunting obstacle for LDNs, who, without differentiated support, may find themselves submerged under unattainable obligations during health crises. Thus, the fate of CBDR, wavering between strengthening and potential exclusion [(11): Article 3.7], becomes integral in dictating the efficacy of the treaty in bridging disparities and fortifying global health security, especially for LDNs.

The lack of specificity and comprehensiveness in the treaty’s stipulations concerning compliance and implementation further accentuates the challenges for LDNs (45). While existing provisions, such as those advocating for the implementation of review mechanisms’ recommendations [(10): Article 13.6] and periodic reporting to the Governing Body [(10): Article 22.2], lay a fundamental groundwork, they fall short of ensuring stringent adherence and accountability, particularly in varied national contexts like those of LDNs. Given their often-limited resources and infrastructural capabilities, LDNs could find themselves navigating a treacherous path through the treaty’s expectations and obligations, potentially jeopardizing their access to essential resources and support during health crises. The absence of a robust mechanism to address non-compliance and resolve disputes, along with a lack of detailed incentives or sanctions to foster adherence (46, 47), suggests that the treaty, in its current form, may inadvertently perpetuate health disparities rather than mitigate them, emphasizing a critical need for refinement to genuinely support LDNs in future pandemics.

Although the proposed pandemic treaty ostensibly offers LDNs enhanced representation and participation, particularly through inclusive decision-making processes and new stakeholder consultation bodies, there linger substantive concerns regarding sovereignty and autonomy that warrant critical examination (3, 38). Notwithstanding the treaty’s affirmation of the sovereign right of each Party to manage public health matters in line with its national legislation and policies [(10): Article 4.3], it delineates obligations and standards that may inadvertently impinge upon the autonomy of LDNs in shaping their health policies and priorities. For instance, while Article 10 mandates sharing biological materials, genetic data, and benefits arising from them (10), it lacks explicit safeguards to ensure that such sharing is predicated on the consent of LDNs and involves equitable benefit-sharing (48). Furthermore, the treaty, while specifying that the Governing Body shall consist of representatives from all Parties [(10): Article 20.1], subject to alternative arrangements in exceptional circumstances [(10): Article 20.3], does not detail the mechanisms of selecting these representatives nor guaranteeing that the unique challenges and contexts of LDNs are aptly reflected in governance processes. Moreover, the absence of mechanisms to ensure robust participation and representation of various stakeholders, including civil society and local health workers, in treaty governance processes, potentially leaves the insights and expertise of those on the frontlines of health crises in LDNs unheard (3). Thus, while the treaty provides a foundational framework to incorporate LDN perspectives into global health governance, the potential pitfalls in safeguarding their sovereignty and autonomy, especially in terms of resource sharing and governance participation, necessitate meticulous refinement to ensure that the treaty not only provides a seat at the table for LDNs but also genuinely empowers them to influence global health policy and practice in a manner that safeguards their interests and autonomy.

### Recommendations to amplify the treaty’s impact on LDNs

To make the pandemic treaty more effective for LDNs, some specific changes and additions to the existing draft are necessary. First, it is vital to revise Article 6 (10) to guarantee that LDNs have prioritized access to vital resources like vaccines and treatments during health crises. This could mean setting a minimum allocation of resources for LDNs. In terms of ensuring that all nations follow the treaty, it is crucial to add clear compliance mechanisms to Article 13.6 (10) or introduce a new article. This might involve specifying penalties for non-compliance and creating a body to manage disputes and review compliance, ensuring all nations are held accountable and that the treaty is implemented fairly. When looking at Article 8 (10), protecting LDNs’ autonomy in sharing resources and information is key. The article should be edited to include mechanisms that ensure LDNs can share pathogens and genetic data consensually and equitably (49). Also, a clear system for sharing benefits, like access to innovations developed using shared resources, is essential to protect LDNs’ interests.

Adding a dedicated fund to the treaty, perhaps in Article 19 (10) or a new article, will also be crucial to financially support LDNs’ pandemic response efforts (50). This fund should be supported by mandatory contributions from wealthier countries and managed transparently, with LDNs having a say in how funds are allocated and used. Including provisions in the treaty that commit to exploring and implementing debt relief or restructuring programs for LDNs is also necessary. This would allow them to reallocate financial resources to public health and pandemic preparedness, ensuring they have the necessary infrastructure to manage health crises effectively.

Engaging the private sector through the treaty is also crucial (51). This means including provisions that encourage partnerships and investment in LDNs’ healthcare infrastructure, perhaps by offering incentives like tax breaks for companies that invest in these areas. The treaty should also mandate the creation of a central knowledge hub, possibly under the management of the WHO, which would share best practices and guidance tailored to LDNs (52). Furthermore, the treaty should encourage the creation of peer networks among LDNs to facilitate knowledge and strategy sharing in pandemic preparedness and response (52).

Lastly, the treaty should require comprehensive needs assessments in LDNs to identify gaps in healthcare infrastructure, workforce, and logistics. Based on these assessments, capacity-building programs can be developed and implemented to ensure LDNs are adequately supported and empowered to effectively respond to pandemics.

## Conclusion

As the global community grapples with navigating and preparing for future pandemics, the emphasis on crafting a potent pandemic treaty is both timely and imperative. However, the unique circumstances, challenges, and strengths of LDNs must be central to these negotiations as we progress toward a comprehensive global solution. The COVID-19 pandemic starkly revealed the structural vulnerabilities of LDNs, underscoring the urgency of shaping a treaty that is comprehensive in approach and grounded in principles of equity and justice.

In its preliminary form, the proposed pandemic treaty undoubtedly promises to shape a more coordinated and effective global response to future health crises. Yet, as elucidated in this analysis, profound gaps and challenges that could marginalize LDNs remain and demand precise and committed resolutions. The recommendations offered provide a pragmatic path to ensuring that, when finalized, the treaty truly empowers LDNs, affording them not only the right to equitable access to resources but also amplifying their voice in global health governance.

In a broader context, the treaty provides an unprecedented opportunity to reimagine and reshape international health architecture beyond historic inequalities and power imbalances. Focusing on LDNs is not just a matter of altruism or international solidarity; it is a recognition of the concept that the health security of one nation inevitably influences the collective in an interconnected world. Ensuring that LDNs are equipped, represented, and heard in this global endeavor is a moral imperative and a pragmatic strategy for fostering a more resilient and inclusive global health ecosystem.

This paper aspires to spark a more informed and inclusive dialogue about the pandemic treaty, placing the interests of the most vulnerable at the core of global decision-making. Only through such an approach can the global community genuinely hope to formulate a treaty that stands the test of time, serving not only as a legal instrument but also as a testament to collective commitment and shared responsibility in the face of global health challenges.

## Data availability statement

The original contributions presented in the study are included in the article/supplementary material, further inquiries can be directed to the corresponding author.

## Author contributions

SJ: Conceptualization, Data curation, Formal analysis, Funding acquisition, Investigation, Methodology, Resources, Writing – original draft, Writing – review & editing. EK: Conceptualization, Methodology, Project administration, Validation, Writing – original draft, Writing – review & editing.
